# Ghosts on the Membrane: Cytoskeletal Pinning Influences Nanoscale Cell Membrane Organization

**DOI:** 10.3390/biom16040596

**Published:** 2026-04-17

**Authors:** Shambhavi Pandey, Thorsten Wohland

**Affiliations:** 1 Centre for Bio-Imaging Sciences, National University of Singapore, Singapore 117546, Singapore; shambhavi@u.nus.edu; 2 Department of Biological Sciences, National University of Singapore, Singapore 117543, Singapore; 3 Department of Chemistry, National University of Singapore, Singapore 117543, Singapore

**Keywords:** plasma membrane, cortical actin, cytoskeletal pinning, GPI-anchored proteins, transbilayer coupling, membrane topography, ITIR-FCS, cholesterol

## Abstract

The lateral organization of the plasma membrane (PM) is vital for cellular signaling, yet the specific mechanisms by which the internal cortical actin meshwork templates the organization of the external lipid leaflet remain poorly understood. While established models like the ‘picket-fence’ emphasize physical barriers to diffusion, recent observations of fiber-like “ghost” structures in the distribution of glycosylphosphatidylinositol-anchored proteins (GPI-APs) suggest a more intricate mode of spatial coordination. In this study, we utilize imaging total internal reflection fluorescence correlation spectroscopy (ITIR-FCS) and variable-angle TIRF to resolve whether these filamentous patterns represent genuine membrane-proximal features or optical artifacts of cytosolic transport. Our results demonstrate that these fiber-like tracks are strictly confined to the immediate PM interface and disappear as the evanescent field probes deeper into the cytosol. While the spatial distribution of GPI-APs is templated by the underlying actin meshwork, quantitative diffusion mapping shows that the lateral dynamics of the probe remains largely uniform and is not significantly modulated by these filamentous patterns. By pharmacologically perturbing the actin scaffold and membrane cholesterol, we show that this transbilayer coupling is contingent upon a cholesterol-dependent cytoskeletal pinning mechanism. These findings demonstrate a decoupling of spatial organization and molecular dynamics, providing evidence for how the actin scaffold patterns nanoscale membrane organization without imposing long-range barriers to diffusion.

## 1. Introduction

**Figure 1:**
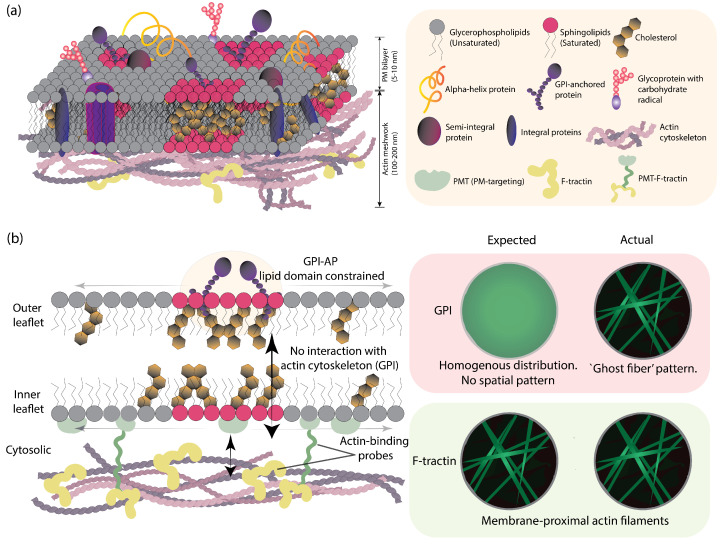
Conceptual framework of membrane-cytoskeleton connection and protein distribution. (**a**) Schematic representation of the plasma membrane interface. Indicated dimensions for the lipid bilayer (∼5–10 nm) and cortical actin thickness (∼100–200 nm) provide the physical context for the TIRF penetration depths used in this study. The inner leaflet is characterized by a dense cortical actin meshwork (pink filaments) and actin-binding proteins like F-tractin (yellow). The outer leaflet contains lipid-anchored proteins (GPI-anchored proteins, purple) and saturated sphingolipids and cholesterol-rich microdomains (magenta clusters). (**b**) Predicted vs. observed spatial distributions for F-tractin and GPI. While F-tractin is expected and observed to form clear, membrane-proximal actin filaments (bottom row), the outer-leaflet GPI is expected to show a homogeneous distribution (top row, left). The actual observation, however, shows the presence of “ghost fiber” patterns in the GPI channel (top row, right), which aligns with the cortical actin cytoskeleton.

The plasma membrane (PM) is a highly complex, dynamic, and heterogeneous interface, serving not only as a semi-permeable barrier but also as a highly organized platform for signal transduction and cellular signaling. Structurally, it is defined by a non-random distribution of hundreds of lipid species across two asymmetric leaflets, creating a distinct physical environment on the exterior versus the cytosolic face (Figure 1a). Composed of a diverse mixture of lipids and proteins, the PM exhibits spatial organization across multiple length scales [1,2,3]. This lateral heterogeneity arises from the collective physicochemical interactions among lipids and membrane proteins, which coordinate to form specialized microdomains that act as functional hubs within the fluid bilayer (Figure 1a) [4,5,6,7]. This compartmentalization has been shown to regulate signaling pathways, molecular diffusion, and protein sorting by locally modulating the composition and physical properties of the membrane [8,9,10,11]. Consequently, understanding how membrane components organize has become a central question in membrane biophysics and cell biology.

Although lipid-driven phase separation provides a thermodynamic basis for membrane heterogeneity, the lateral organization of PM is strongly influenced by the underlying actin cortex [12,13,14]. According to the ‘picket-fence’ model, the membrane-proximal actin meshwork and its associated transmembrane ‘pickets’ act as physical barriers that compartmentalize the membrane into hopping-diffusion corrals [14,15,16].

Previous studies have utilized different membrane probes to characterize these specific confinement modes. For instance, proof-of-principle experiments on CHO-K1 cells have confirmed that different probes represent distinct organizational regimes: the lipophilic dye DiI-C18 typically exhibits free Brownian diffusion, whereas the inner-leaflet probe PMT-GFP (GFP fused to a PM-Targeting sequence) is characterized by actin-dependent hop diffusion, indicating association with the actin cortex [17].

In this context, the behavior of outer-leaflet GFP-GPI-AP (Glycosylphosphatidylinositol-anchored protein) is of particular interest. Lacking an intracellular domain for direct cytoskeletal attachment, GPI is generally associated with cholesterol-dependent domain confinement rather than actin-mediated partitioning [1,17,18,19,20,21]. To visualize this relationship, we utilize the live-cell actin probe F-tractin to provide a high-contrast image of the actin cytoskeleton alongside the GPI distribution (Figure 1a). The spatial organization and dynamics of these two probes should be decoupled: F-tractin is expected and observed to form clear underlying filaments, whereas the lipid-anchored GPI should exhibit a homogeneous or domain-confined distribution on the PM (Figure 1b).

Despite this distinction, recent observations have shown the presence of fiber-like ‘ghost’ structures in the distribution of GPI that appear to align with the underlying actin meshwork [22]. Because GPI lacks an intracellular domain to facilitate direct actin binding and is primarily governed by cholesterol-dependent confinement, these observations raise a fundamental question: do these fiber-like structures represent a coordinated transbilayer templating mechanism, or are they an optical artifact?

One mechanistic possibility is that the cytoskeleton actively templates membrane order through ‘pinning’ [13,23,24,25]. As demonstrated by Honigmann et al. [23], a membrane-bound actin meshwork can organize liquid phase separation in model membranes by acting as pinning sites for order-preferring lipids. In this framework, the cytoskeleton stabilizes liquid-ordered ($L_{o}$) domains by providing a scaffold that prevents their macro-scale coalescence. This pinning is inherently cholesterol-dependent, suggesting that the ‘ghost fibers’ observed in GPI distributions could be emergent structures formed when membrane cholesterol pins the $L_{o}$ phase to the underlying actin template.

Alternatively, these fiber-like patterns may not represent the organization of the PM itself. Because membrane proteins are synthesized and trafficked via intracellular vesicles [26,27], fluorescent signals detected in the TIRF evanescent field may originate from cytosolic transport intermediates residing on actin tracks just beneath the bilayer.

To resolve these competing models, the physical dimensions of the membrane interface must be reconciled with the optical observation volume. The PM bilayer itself spans approximately 5–10 nm, while the underlying cortical actin meshwork typically extends 100–200 nm into the cytosol (Figure 1a) [28,29]. Furthermore, the gap between the PM and the dense cortical layer is estimated at ∼10–20 nm [30]. Because the TIRF evanescent field typically penetrates 80–200 nm into the sample, it inherently samples a volume that encompasses the bilayer, the membrane-cortex gap, and a significant portion of the cortical scaffold. Understanding these spatial constraints is critical for distinguishing genuine membrane-localized ’pinning’ from signals originating from sub-membranous cytosolic structures.

In this work, we employ Imaging Total Internal Reflection-Fluorescence Correlation Spectroscopy (ITIR-FCS). Unlike conventional single-point FCS, ITIR-FCS utilizes TIR illumination to selectively excite the membrane-proximal environment. By modulating the TIRF incident angle, we vary the TIRF evanescent field or the penetration depth from 80 nm to 160 nm. This range is chosen to transition from a regime dominated by the PM and immediate cortex (∼5–10 nm and ∼100 nm thick, respectively). This approach provides the high signal-to-noise ratio (SNR) necessary to analyze fluorescence fluctuations at thousands of contiguous spots while minimizing interference from the bulk cytosol [31,32]. This method generates spatially resolved parametric maps, primarily diffusion maps (*D*-maps), which represent the lateral mobility of probes at every pixel across the imaging field. By combining this quantitative mapping with variable-angle TIRF to modulate penetration depth and pharmacologically perturbing both the actin cytoskeleton and cholesterol, we aim to resolve how the actin scaffold dictates the lateral spatial organization and dynamics of the PM.

## 2. Materials and Methods

### 2.1. Cell Culture

CHO-K1 cells (CCL-61™, ATCC; Manassas, VA, USA) were cultured in Dulbecco’s Modified Eagle Medium (DMEM, high glucose with L-glutamine, without sodium pyruvate; SH30022.FS, HyClone, GE Healthcare Life Sciences, Salt Lake City, UT, USA). The medium was supplemented with 10% fetal bovine serum (FBS; #10270106, Gibco, Thermo Fisher Scientific, Waltham, MA, USA) and 1% penicillin–streptomycin (#15070063, Gibco). Cells were maintained in 25 cm^2^ polystyrene culture flasks (#430639, Corning, NY, USA) and passaged upon reaching confluency. Cultures were incubated at 37 °C in a humidified atmosphere containing 5% (*v*/*v*) CO_2_ using a CO_2_ incubator (Forma Steri-Cycle, Thermo Fisher Scientific).

### 2.2. Plasmids and DiI-C18 Staining

The plasma membrane probe PMT-mEGFP (Addgene #203777) has been described previously [33]. The GFP-GPI plasmid was kindly provided by Dr. John Dangerfield (Anovasia Pte Ltd., Singapore). Construction of PMT-mEGFP-F-tractin and F-tractin-mEGFP plasmids has been described in [22].

For membrane staining, stock solution of DiI-C18 prepared in DMSO was diluted in Hank’s Balanced Salt Solution (HBSS) to a final concentration of 100 nM. Culture medium was removed from seeded cells, and the DiI-C18 working solution was added, followed by incubation at 37 °C for 15 min. Cells were subsequently washed twice with HBSS to remove excess dye. Finally, 1 mL of HBSS was added to the cells prior to imaging.

### 2.3. Cell Transfection

CHO-K1 cells at approximately 90% confluence were used for transfection. Cells were washed twice with phosphate-buffered saline (PBS) without Ca^2+^ and Mg^2+^. Subsequently, 2 mL of TrypLE™ Express enzyme (Gibco, Thermo Fisher Scientific) was added, and cells were incubated at 37°C for 2–3 min to facilitate detachment. The enzymatic reaction was stopped by adding 5 mL of complete culture medium, followed by centrifugation at 200× *g* for 3 min.

The cell pellet was resuspended in PBS and counted using an automated cell counter (TC20, Bio-Rad, Hercules, CA, USA). Approximately 4–5 × 10^5^ cells were pelleted and resuspended in R buffer (Neon Transfection Kit, Thermo Fisher Scientific). For each transfection, 100 ng of plasmid DNA was mixed with the cell suspension. Transfection was performed using Lipofectamine™ 3000 (Invitrogen™, Thermo Fisher Scientific, Carlsbad, CA, USA) according to the manufacturer’s protocol.

Transfected cells were seeded onto glass-bottom culture dishes (#P35G-1.5-20-C, MatTek, Ashland, MA, USA) containing DMEM supplemented with 10% FBS (without antibiotics) and incubated at 37 °C with 5% CO_2_ for 36–48 h prior to imaging.

### 2.4. Sample Preparation for Fluorescence Measurements

Prior to measurements, cells were rinsed with HBSS (with Ca^2+^ and Mg^2+^; #14025134, Gibco). Imaging was performed in phenol-red-free DMEM (#21063029, Gibco), referred to as ‘Imaging DMEM’ throughout this study.

### 2.5. Drug Treatments

Drug working solutions were prepared using Imaging DMEM. To modulate actin cytoskeleton dynamics, cells were treated with 1 μM Jasplakinolide (Jas; #J7473, Sigma-Aldrich, St. Louis, MO, USA) to promote actin polymerization or 1 μM Latrunculin A (Lat-A; #L5163, Sigma-Aldrich) to disrupt actin filaments. Cells were incubated with the respective drugs for 30 min prior to imaging.

For cholesterol depletion experiments, methyl-*β*-cyclodextrin (M*β*CD; Sigma-Aldrich) was used. A 100 mM stock solution was prepared according to the manufacturer’s protocol (#C4555). Cells were washed with HBSS and incubated with 3 mM M*β*CD for 30 min, followed by rinsing with HBSS and replacement with Imaging DMEM before measurements.

### 2.6. Instrumentation

Total internal reflection fluorescence (TIRF) microscopy measurements were performed using an inverted epifluorescence microscope (IX83, Olympus, Singapore) equipped with a PlanApo 100× oil-immersion objective (NA 1.49, Olympus) and a back-illuminated EMCCD camera (iXON 860, 128 × 128 pixels; Andor Technology, Belfast, UK).

Excitation was provided by 488 nm and 561 nm lasers (LAS/488/100 and LAS/561/100, Olympus), coupled through a TIRF illumination combiner (cellTIRF/IX3-MITICO, Olympus). A quad-band dichroic mirror (ZT 405/488/561/640rpc, Chroma Technology Corp., Rockingham, VT, USA) was used to direct the excitation light into the objective. Fluorescence emission was filtered using a quad-band emission filter (ZET405/488/561/640m, Chroma Technology Corp.) before detection.

The angle of incidence was adjusted using Olympus cellSens imaging software (version 3.1.1, Olympus Corporation, Tokyo, Japan) to achieve TIRF illumination. Measurements were performed at 37 °C and 5% CO_2_ using an on-stage incubator (Chamlide TC, Live Cell Instrument, Seoul, Republic of Korea).

### 2.7. Data Acquisition

Laser powers ranging from 100 μW to 1 mW (measured at the back focal plane of the objective) were used depending on the expression level of the fluorescent proteins. Unless otherwise specified, a penetration depth of 80 nm was used.

Image acquisition was performed using Andor Solis software (v4.31.30037.0). The EMCCD camera was maintained at −80 °C and operated in kinetic mode with baseline clamp enabled to minimize baseline fluctuations. The camera was set to a pixel readout rate of 10 MHz, an analog-to-digital gain of 4.7, and a vertical shift speed of 0.45 μs. An EM gain of 300 was used.

For each measurement, image stacks consisting of 50,000 frames (128 × 128 pixels) were acquired at a frame rate of 500 frames s^−1^ (2.06 ms per frame). The image stacks were stored in 16-bit TIFF format.

### 2.8. ITIR-FCS Analysis

Image stacks were analyzed using the ImFCS 2.04 plugin in Fiji [34,35]. The parameters used were: frame time = 0.00206 s, pixel size = 24 μm, numerical aperture = 1.49, $\lambda_{1}$ = 510 nm for the green channel and $\lambda_{1}$ = 590 nm for the red channel. The multiple-tau correlator settings were (p,q)=(16,9). Here, *q* represents the number of groups with uniform lag time spacing, while the lag time doubles from group to group. *p* denotes the number of points within the first group, while all subsequent groups have p/2=8 bins of uniform lag time spacing. This architecture enables efficient computation of the ACF over a wide range of lag times while maintaining a high SNR ratio at longer lags [36,37,38].

EMCCD data were analyzed using 1 × 1 pixel binning at 100× magnification. Photobleaching correction was performed using a sixth-order polynomial fit. The autocorrelation functions (ACFs) were fitted with a one-component two-dimensional diffusion model [32]. Temporal binning of the entire stack was performed to obtain a TIRF-averaged image. To ensure high statistical reliability and exclude noise-dominated pixels or immobile fractions, only ACFs that successfully converged to the diffusion model within a strict threshold of 0.01–3 μm^2^/s were included in the final analysis. Global *D* values for each condition were calculated by averaging the valid pixels across multiple independent cells (*n*) for each experimental group.

### 2.9. Statistical Analysis

Diffusion coefficients are reported as mean ± standard deviation (SD). Statistical analyses were performed using Python (version 3.12.13). Differences between conditions were evaluated using two-way analysis of variance (ANOVA), followed by post-hoc tests where appropriate. Statistical significance was defined as *** p<0.001, ** p<0.01, * p<0.05, and n.s. for non-significant differences. Data visualization and statistical annotation were performed using the Matplotlib and Seaborn libraries in Python.

## 3. Results

### 3.1. Observation of Filamentous Membrane Patterns Mirroring the Actin Cortex

**Figure 2:**
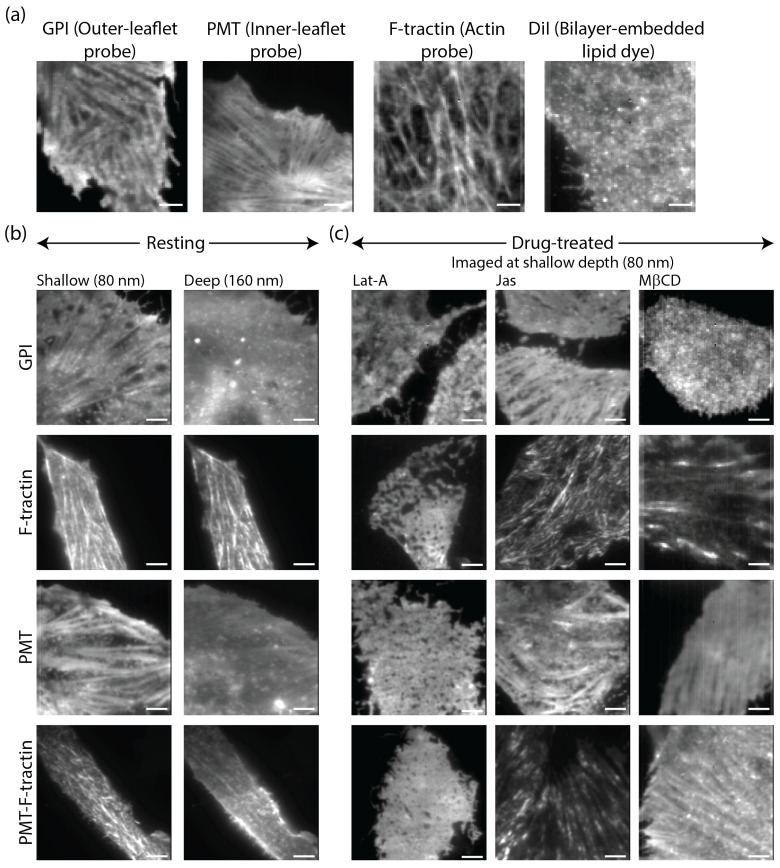
Membrane probes exhibit actin-aligned fiber-like patterns in TIRF microscopy. (**a**) Representative TIRF images (penetration depth = 80 nm) of cells expressing outer leaflet probe GPI, inner leaflet probe PMT, actin probe F-tractin, and lipophilic membrane dye DiI-C18. (**b**) Comparison of membrane patterns at different TIRF penetration depths (80 nm vs. 160 nm) for GPI, PMT, F-tractin, and the PMT–F-tractin combination. Display contrast for individual images has been adjusted to facilitate the visualization of structural morphology at different depths. (**c**) Effects of cytoskeletal perturbation (Lat-A, Jas) and cholesterol depletion (M*β*CD) on membrane organization, measured at 80 nm penetration depth. Scale bar = 5 μm.

To map the spatial relationship between the PM and the actin cortex, we utilized TIRF microscopy with a shallow penetration depth (80 nm). The actin-binding probe F-tractin showed a dense network of filamentous structures consistent with the known architecture of the cortical actin meshwork (Figure 2a). Surprisingly, we observed similar fiber-like structures when imaging both the inner-leaflet probe PMT and the outer-leaflet probe GPI (Figure 2a). These structures have been shown to be aligned along the actin fibers [22]. This observation is significant because these probes represent distinct biochemical identities and confinement regimes. While PMT is associated with the actin cytoskeleton, GPI is primarily governed by cholesterol-dependent domain confinement and lacks a direct intracellular domain for cytoskeletal attachment [17,39]. To test if this filamentous patterning is a universal feature of membrane labeling, we imaged cells stained with the lipophilic dye DiI-C18. Unlike the protein-anchored probes, DiI exhibited a largely homogeneous distribution with no visible filament patterning (Figure 2a).

A critical interpretive challenge in TIRF microscopy of membrane-anchored probes is the presence of a transport pool. Fluorescently labeled proteins, such as GPI, are synthesized and trafficked to the surface via intracellular vesicles that frequently utilize cortical actin filaments as tracks for directed transport. Within the evanescent field, these pre-insertion cytosolic vesicles can appear as bright features aligned along actin fibers, potentially mimicking genuine PM organization. To resolve whether the ‘ghost fibers’ observed at 80 nm (Figure 2a) represent true membrane templating or a mere shadow of this cytosolic transport machinery, we performed variable-angle TIRF to selectively increase the evanescent wave penetration depth.

By probing deeper into the cytosol, we could distinguish between signals originating from the bilayer and those from the sub-membrane space. As the penetration depth increased to 160 nm, we observed a distinct divergence in the structural clarity of the fibers we observed at shallow depth. While actin-binding markers, F-tractin and PMT-F-tractin, maintained their prominent filamentous morphology across the two measured depths, the fiber-like patterns for GPI were rapidly eliminated (Figure 2b). Notably, the inner-leaflet probe PMT continued to show discernible fiber-like structures even at 160 nm (Figure 2b), albeit with lower contrast than at 80 nm. This persistence in the PMT channel, which contrasts with the complete loss of structure in the GPI channel, likely reflects the inherent association of the PMT sequence with the actin-rich cortex.

At deeper penetration depth, the signal from the GPI transitioned from a structured, fiber-aligned state to a punctate or clustered distribution. While the representative images in Figure 2b are contrast-adjusted to highlight this structural transition, the fluorescence intensity of the membrane-proximal GPI undergoes significant decay as the evanescent field probes deeper into the cytosol, quantitatively explored in the subsequent sections via intensity distribution analysis. This depth-dependent loss of filamentous morphology indicates that the fiber structures observed at 80 nm are not the result of a cytosolic pool of GPI residing on actin filaments. Instead, these structures are restricted to the immediate vicinity of the PM, confirming that the observed organization is a membrane-proximal phenomenon.

To further investigate the dependency of these membrane patterns on the underlying cytoskeleton, we pharmacologically perturbed actin composition (Figure 2c). Stabilization of the actin network with 1 μM Jasplakinolide (Jas) resulted in a marked enhancement and strengthening of the fiber-like patterns in both the GPI and PMT channels (Figure 2c). This was accompanied by a notable increase in the prominence and density of the actin meshwork as visualized by F-tractin and PMT-F-tractin (Figure 2c). Conversely, depolymerization of the actin meshwork using 1 μM Latrunculin A (Lat-A) completely abolished these “ghost” structures, reverting the lipid distribution to a diffuse state (Figure 2c). As expected, this loss of membrane patterning coincided with the near-total disappearance of fiber structures in the F-tractin and PMT-F-tractin channels, confirming that the lipid-based fibers are contingent upon a polymerized actin scaffold (Figure 2c).

Following the hypothesis that the cytoskeleton acts as a pinning site for membrane domains [23], we utilized a mild concentration of methyl-*β*-cyclodextrin (M*β*CD, 3 mM) to reduce membrane cholesterol. At this concentration, the global actin cytoskeleton (visualized via F-tractin) remained largely intact (Figure 2c), showing no obvious structural changes. However, the fiber-like patterns in the GPI channel were lost, and the PMT channel showed a significant reduction in fiber definition (Figure 2c). These findings suggest that the alignment of membrane components along the actin tracks is highly sensitive to cholesterol abundance, even when the underlying cytoskeletal architecture remains unperturbed. This points toward a potential role for cholesterol as a mediator in the coupling between the membrane bilayer and the cortical scaffold.

### 3.2. Depth-Dependent Analysis of Probe Dynamics and Structural Persistence

**Figure 3:**
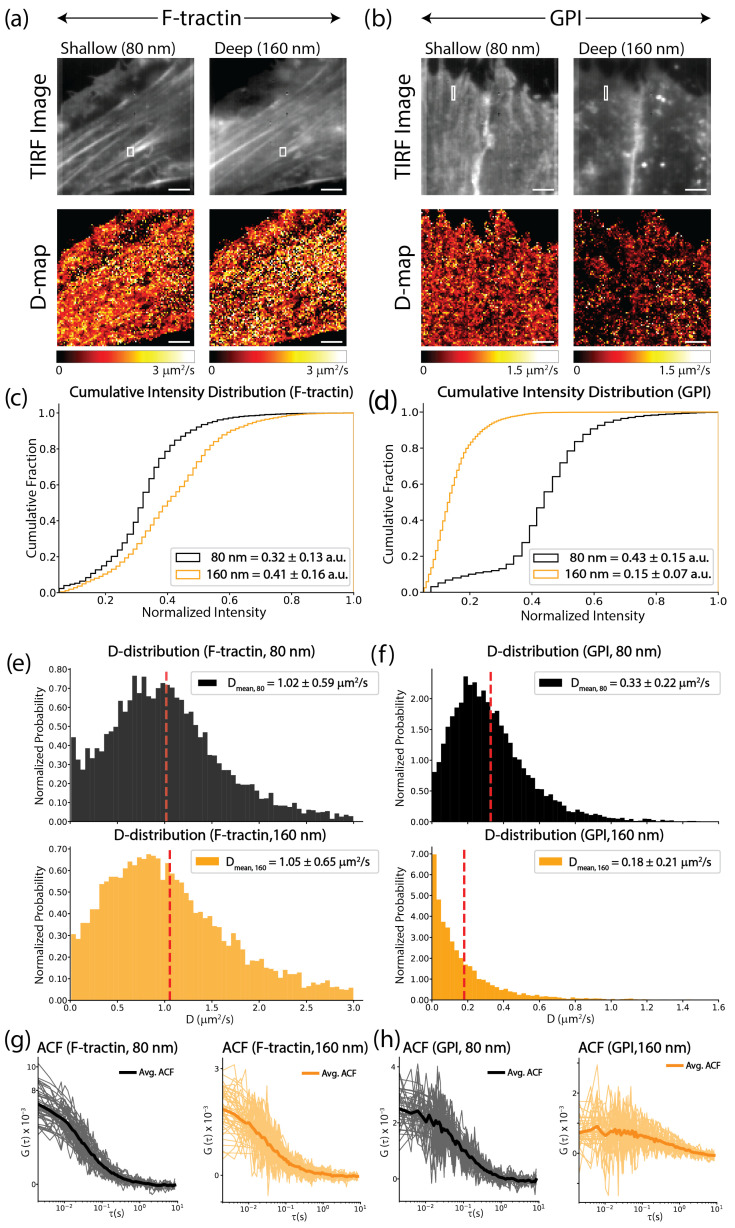
Quantitative discrimination of membrane vs. cytosolic probe dynamics. (**a**,**b**) Representative TIRF microscopy images and corresponding ImFCS-derived diffusion coefficient (*D*) maps for cells expressing (**a**) F-tractin and (**b**) GPI, captured at shallow (80 nm) and deep (160 nm) penetration depths. White boxes indicate representative regions selected for localized autocorrelation analysis. (**c**,**d**) Cumulative fluorescence intensity distributions for (**c**) F-tractin and (**d**) GPI for the cells shown in (**a**,**b**). Black curves represent the intensity distribution at 80 nm, and orange curves represent 160 nm. (**e**,**f**) Frequency distributions of diffusion coefficients (*D*) for (**e**) F-tractin and (**f**) GPI corresponding to the maps in (**a**,**b**). Red dashed lines indicate the mean diffusion coefficient for each distribution. (**g**,**h**) Representative ImFCS Autocorrelation Functions (ACF) extracted from the fiber regions marked in (**a**,**b**) for (**g**) F-tractin and (**h**) GPI. Black curves represent 80 nm and orange curves represent 160 nm penetration depths. The background curves represent individual pixel ACFs, while the bold solid line represents the average ACF. Scale bar = 5 μm. Inset numerical data represent mean ± SD.

To quantify the depth-dependent loss of fiber-like organization observed in Figure 2, we performed a comprehensive analysis of the fluorescence intensity and molecular dynamics for both actin (F-tractin) and lipid (GPI) probes. We first examined the time-averaged TIRF images and the corresponding ITIR-FCS-derived diffusion coefficient (*D*) maps (Figure 3a,b). Visually, F-tractin maintained a robust filamentous architecture and consistent *D*-map appearance at both 80 nm and 160 nm penetration depths (Figure 3a). In contrast, while GPI exhibited clear fiber-like patterns and uniform dynamics at the shallow 80 nm depth, these features largely disintegrated at 160 nm, leaving behind a sparse, punctate signal with highly heterogeneous and slower diffusion (Figure 3b).

These visual changes were supported by the cumulative intensity distributions. The probability distribution of F-tractin intensities remained stable between depths, reflecting consistent labeling of the cortical meshwork throughout the evanescent field (Figure 3c). However, the intensity distribution for GPI shifted significantly toward lower values as penetration depth increased (Figure 3d). Specifically, the mean intensity (〈I〉) for F-tractin increased slightly from 0.32±0.13 a.u. to 0.41±0.16 a.u. at 160 nm due to deeper cortical sampling, while the GPI signal dropped significantly from 0.43±0.15 a.u. at the surface to 0.15±0.07 a.u. at the deeper depth.

This difference was further quantified by assessing the global *D*-distributions across the entire cell. The mobility profile for F-tractin remained consistent regardless of the penetration depth (Figure 3e). Conversely, the *D*-distribution for GPI showed a marked shift and broadening as the sampling depth increased to 160 nm (Figure 3f), yielding a coefficient of variation (CoV, defined by standard deviation/mean) exceeding 110% at 160 nm depth for this representative cell. To understand the origin of this degradation, we examined the Autocorrelation Functions (ACFs) specifically within the fiber-rich regions (Figure 3g,h) marked in the TIRF images of the cell (Figure 3a,b). While F-tractin ACFs showed high SNR (Figure 3g), the ACFs for GPI at 160 nm failed to resemble standard membrane diffusion profiles (Figure 3h). Collectively, these results confirm that the organized diffusion and fiber-like patterning of GPI are membrane-proximal phenomena that exist only when the probe is constrained at the PM interface.

### 3.3. Depth-Dependent Dynamics and Pharmacological Perturbation of Membrane-Actin Coupling

**Figure 4:**
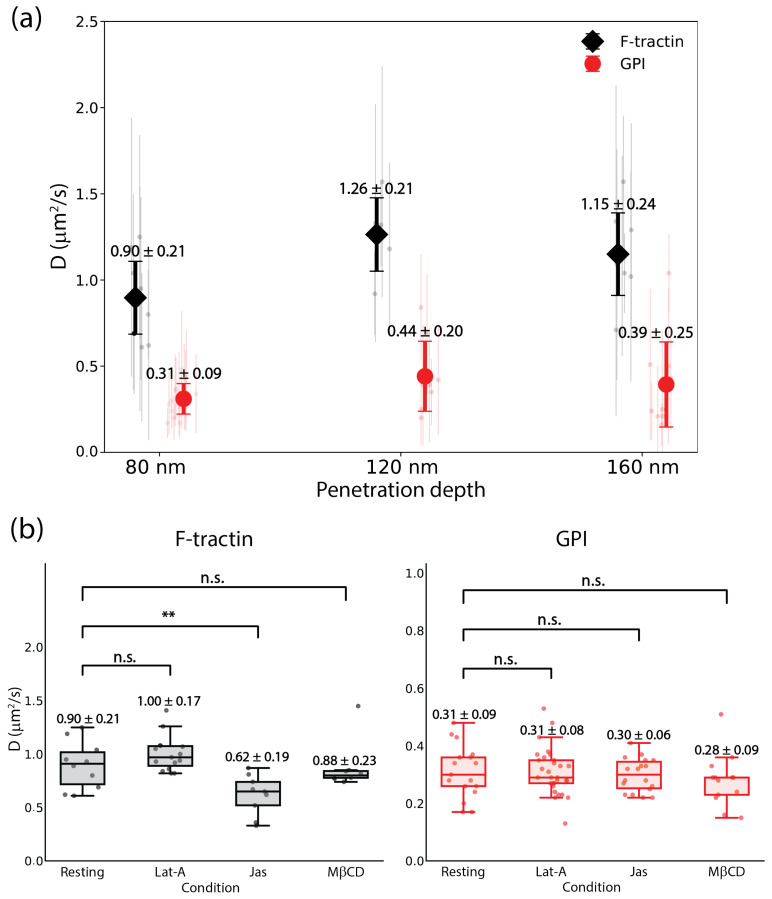
Depth-dependent and pharmacological modulation of probe dynamics. (**a**) Global diffusion coefficients (*D*) for F-tractin (black diamonds) and GPI (red circles) as a function of TIRF penetration depths (80 nm, 120 nm and 160 nm). Individual cell measurements are shown as faded points with the global mean ± SD overlaid. (**b**) Effect of pharmacological perturbations on molecular dynamics at a fixed penetration depth of 80 nm. F-tractin (left) and GPI (right) diffusion coefficients are shown for resting cells and those treated with 1 μM Latrunculin-A (Lat-A), 1 μM Jasplakinolide (Jas), and 3 mM methyl-*β*-cyclodextrin (M*β*CD). Statistical significance was defined as ** p<0.01, and n.s. for non-significant differences.

To investigate the spatial extent of the observed fiber visualization with GPI, we analyzed the measured dynamics of F-tractin and GPI across a range of penetration depths from 80 nm to 160 nm. This range allows for quantitative diffusion mapping of the transition from the immediate membrane-proximal zone at shallow depths (80–100 nm) to the sub-cortical environment (100–160 nm). To maintain high statistical reliability, the analysis exclusively utilized ACF fits within a diffusion coefficient threshold of 0.01–3 μm^2^/s. The global *D* values summarized in Figure 4a and Table 1 show how these probes experience the cellular environment as a function of depth.

For the actin probe, F-tractin exhibited its lowest dynamics at the 80 nm surface with a *D* of 0.90±0.21 μm^2^/s. As the sampling volume extended deeper into the cell, the mobility increased to 1.26±0.21 μm^2^/s at 120 nm, reflecting a physical transition from the highly constrained and dense cortical meshwork to more mobile, less cross-linked actin filaments in the deeper cytosol (Figure 4a). At even deeper penetrations of 140 nm and 160 nm, the reported *D* values stabilized or slightly decreased to 1.18±0.33 μm^2^/s and 1.15±0.24 μm^2^/s, respectively. This minor decrease in the 140–160 nm range is likely an artifact of sampling deeper cytosolic volumes, accompanied by lower ACF amplitudes and significantly higher individual standard deviations as the SNR decreases (Table 1).

In contrast, the membrane-anchored GPI probe maintained a consistent diffusion profile across all measured depths, ranging from 0.31±0.09 μm^2^/s at the surface to 0.39±0.25 μm^2^/s at 160 nm. However, while the ensemble mean remains stable, the underlying pixel-wise distributions of individual cells exhibit increased variance at 160 nm. This is illustrated in the representative cell in Figure 3f, where the CoV% increases to 110% at the deepest penetration. When pooled across all independent experiments (n≥10), this trend persists with the average CoV% increasing from 29.0% at the surface to 64.1% at 160 nm (Table 1). This increase in variance corresponds to the reduction in SNR at deeper penetration depths, which leads to degraded ACF quality and reduced precision in the individual fits. The consistency of the mean *D* in Table 1 is a result of ensemble averaging and the inclusion of only those pixels yielding ACFs that successfully converged within the set threshold. Crucially, as the evanescent field sampled a larger sub-cortical volume at 160 nm, we observed a significant increase in the standard deviation of diffusion coefficient obtained with GPI (Figure 4a). This increase in variance, combined with the observed degradation of ACF quality, indicates that the structured fiber-like diffusion is a phenomenon strictly confined to the immediate vicinity of the PM and has no significant effect on the global *D* values reported for GPI. These findings highlight a significant decoupling between spatial organization and global transport; while the structural templating of GPI into fiber-like tracks is lost beyond the shallow depth illumination zone, the intrinsic dynamics of the molecules remains largely unaffected, suggesting the actin cortex acts as a spatial template rather than a global confinement barrier.

To further dissect the nature of the observed membrane-actin coupling, we evaluated the diffusion of both probes under specific pharmacological perturbations at a fixed penetration depth of 80 nm (Figure 4b). The resulting *D* values for F-tractin and GPI across resting and treated states are summarized in Table 2.

For F-tractin, the application of 1 μM Jas, which stabilizes actin filaments, resulted in a significant reduction of F-tractin mobility to 0.62±0.19 μm^2^/s. The polymerization of the network confirms that stabilizing the actin meshwork increases the steric hindrance experienced by the probe. Conversely, depolymerizing the network with 1 μM Lat-A led to a slight increase in mobility to 1.00±0.17 μm^2^/s, consistent with the breakdown of the stable meshwork into faster-diffusing fragments. To assess the role of the lipid environment, cells were treated with 3 mM M*β*CD to facilitate cholesterol depletion. At this concentration, which is well below the cytotoxic range and is known to have no significant effect on the actin network, F-tractin dynamics remained unchanged (0.88±0.23 μm^2^/s).

Despite the near-elimination of visual fiber patterns observed in our previous imaging results, the mean diffusion coefficient of GPI remained statistically unchanged across all pharmacological treatments (Figure 4b). GPI diffusion remained consistent at approximately 0.31 μm^2^/s, regardless of whether the actin was depolymerized (Lat-A) or the membrane was depleted of cholesterol (M*β*CD). This provides evidence that while these perturbations disrupt the spatial alignment of GPI into ‘ghost fibers’, they do not significantly alter the intrinsic dynamics experienced by individual molecules within the membrane. This decoupling of spatial organization from global diffusion rates points toward a specific templating mechanism where the actin cytoskeleton provides a structural track for organization without acting as a global barrier to molecular diffusion at the PM.

### 3.4. Cholesterol-Mediated Coupling of the Outer Leaflet to the Actin Cortex

**Figure 5:**
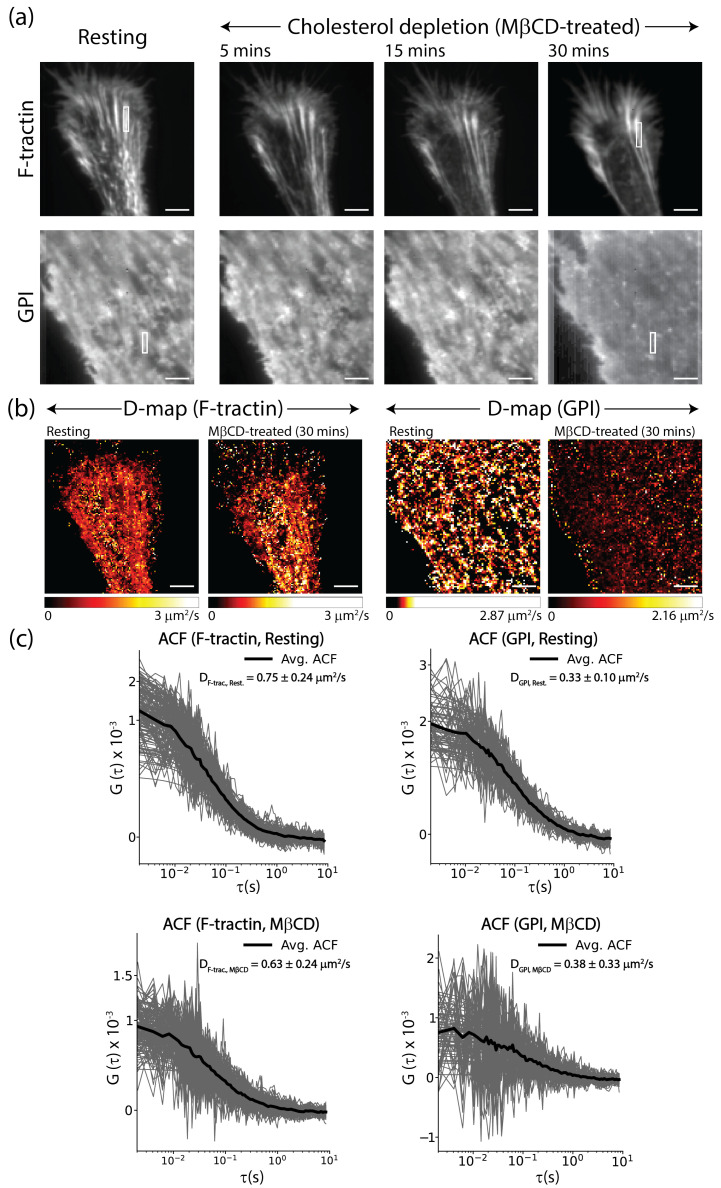
Cholesterol depletion disrupts the spatial templating of GPI without altering actin structure. (**a**) Time-averaged TIRF images of F-tractin (top) and GFP-GPI (bottom) before and after 5, 10 and 30 min of 3 mM M*β*CD treatment. White boxes indicate representative regions selected for localized autocorrelation analysis. (**b**) Corresponding D-maps of F-tractin and GPI before and after 30 min of cholesterol depletion. (**c**) Representative ACFs and D values for F-tractin and GPI before and after cholesterol depletion, for fiber regions as marked in the images shown in (**a**). The background curves represent individual pixel ACFs, while the bold solid line represents the average ACF. Scale bar = 5 μm.

To further investigate the mechanism behind the unexpected fiber visualization of GPI, we studied specifically the role of membrane cholesterol as a potential coupling agent. We compared cells expressing F-tractin and GPI in their resting state and following treatment with 3 mM M*β*CD to induce cholesterol depletion. Imaging results show a striking difference in how the two probes respond to cholesterol depletion over a 30-min period (Figure 5a). The actin fibers remained structurally intact even 30 min post-M*β*CD treatment; although the cell morphology exhibited some drifting and minor changes, the primary filamentous network labeled by F-tractin was preserved (Figure 5a). In contrast, the fiber-like patterns observed with GPI began to disintegrate or visually disappear by the 30-min mark, indicating that the spatial alignment of the outer-leaflet probe along the actin template is lost upon the removal of cholesterol (Figure 5a).

Corresponding D-maps and ACF analyses quantified this transition by focusing on the observed fiber regions (Figure 5b). For F-tractin, the ACF quality and signal amplitude remained consistent, with the diffusion coefficient staying nearly similar, transitioning from 0.75±0.23 μm^2^/s in the resting state to 0.63±0.24 μm^2^ post-depletion (Figure 5c). However, for GPI, the ACF quality and amplitude changed significantly after treatment; while the mean diffusion rate showed a slight increase from 0.33±0.10 μm^2^ to 0.38±0.33 μm^2^, the increase in standard deviation was notable (Figure 5c). This increase in variance likely reflects the transition from a structured, constrained distribution to a more randomized, homogeneous one.

These results show that while cholesterol does not globally alter the dynamics of GPI, the specific visualization of actin-aligned structures at the PM interface is critically dependent on cholesterol. Our observations support a model where cholesterol-mediated pinning acts as the bridge between the outer leaflet and the underlying actin cytoskeleton. The visualization of ‘ghost fibers’ is therefore a consequence of this spatial pinning, whereas the global diffusion rates remain largely unaffected due to the relatively weak influence of the cortex on molecular transport.

## 4. Discussion

A persistent challenge in membrane imaging is the potential for optical crosstalk between the PM and the underlying cytoplasm. Because membrane-anchored proteins like GPI are trafficked via intracellular vesicles that utilize actin tracks, fluorescent signals detected in TIRF could easily be mistaken for genuine membrane organization. Our variable-angle TIRF approach addresses this ambiguity by exploiting the depth-sensitivity of the evanescent field. The observation that F-tractin maintains structural definition while the GPI ‘ghost fibers’ dissipate at larger penetration depths (160 nm) provides a definitive spatial fingerprint. This divergence confirms that the filamentous patterning of GPI is an emergent property of the membrane interface rather than a projection of deeper cytosolic transport intermediates.

One of the most striking findings of this study is the apparent decoupling between the spatial arrangement of GPI and its global diffusion coefficients. While the actin cortex clearly templates the spatial distribution of outer-leaflet probes, it does not act as a pervasive viscous barrier. This suggests a refinement of the classic “picket-fence” model; whereas the model traditionally emphasizes the restriction of lateral mobility through physical barriers, our data indicates that the cytoskeleton can act as a spatial scaffold without necessarily imposing strong kinetic confinement. The stability of $D_{GPI}$ across varying penetration depths and pharmacological actin perturbations suggests that while the ‘ghost fibers’ define where the molecules are, they do not dictate how fast they move within the fluid bilayer.

Our observation of spatially uniform diffusion despite structural patterning is consistent with recent high-resolution mapping studies using super-resolution microscopy-associated diffusion mapping (SMdM) [40]. For instance, Yan et al. showed that while the membrane exhibits nanoscale diffusional heterogeneities, these are primarily driven by local lipid packing order and ER-PM contact sites rather than global modulation by the actin cytoskeleton [40]. In contrast, single-particle tracking (SPT) studies in specialized cellular architectures, such as the periodic actin rings in neuronal axons, show that membrane proteins can be significantly confined between actin filaments [41]. This apparent discrepancy shows the importance of the biological context and the spatiotemporal scales probed. While the dense, periodic actin rings in neurons provide a clear physical barrier to long-range motion, our results in CHO-K1 cells suggest a templating regime. Here, the cytoskeleton templates the spatial distribution of GPI-APs through sparse or transient pinning, but these sites do not form the impenetrable, continuous barriers required to partition the membrane into discrete kinetic compartments.

The sensitivity of the observed patterns to M*β*CD provides mechanistic insight into the transbilayer communication between the inner-leaflet actin and outer-leaflet GPI. The fact that cholesterol depletion eliminates membrane fibers alignment of membrane proteins while leaving the actin meshwork intact points toward a lipid-mediated coupling mechanism. These results are consistent with the ‘cytoskeletal pinning’ hypothesis, where actin filaments serve as nucleation sites for $L_{o}$ phases. In this framework, cholesterol-rich microdomains, which prefer the $L_{o}$ environment, are pinned to the underlying actin template [23,42].

Cholesterol is the essential mediator of transbilayer coupling, physically linking the organization of the two leaflets through inter-leaflet lipid chain interdigitation or via transmembrane ‘picket’ proteins that span the bilayer [43,44,45]. Specifically, cholesterol-rich microdomains in the outer leaflet may be thermodynamically coupled to ordered lipid clusters or picket proteins in the inner leaflet that are directly anchored to the actin scaffold [42,43,44,46]. This pinning creates a spatial template of the cytoskeleton in the outer leaflet, explaining how a probe without a cytosolic domain can perfectly mirror the cortical meshwork.

From a methodological standpoint, our results underscore the necessity of high SNR and precise depth control in Imaging FCS. The observed increase in the variance of diffusion coefficient values and the degradation of ACFs at larger penetration depths show how background noise from the bulk cytosol can obscure membrane dynamics. By combining variable-angle TIRF with spatially resolved *D*-maps, we show a framework for disentangling membrane-proximal organization from deeper cytosolic processes.

While dual-color correlation techniques like simultaneous dual-wavelength imaging or Fluorescence Cross-Correlation Spectroscopy (FCCS) are powerful for detecting the co-diffusion of molecular complexes, their application to the actin-membrane interface is limited by the disparate dynamics of the two components [47,48]. Because the actin cortex remains relatively static on the timescale of membrane diffusion, a diffusing GPI-AP probe that ‘grazes’ or is transiently pinned by a stationary actin filament would not yield a strong cross-correlation signal. Future methodological advances might instead focus on single-molecule co-localization or STED-FCS to resolve the interaction lifetimes of these transient pinning events at higher spatial resolutions.

In summary, our data supports a model where the cortical actin cytoskeleton patterns the PM through a cholesterol-dependent spatial templating mechanism. Rather than acting as a series of rigid corrals, the actin cortex provides a stabilizing scaffold that organizes membrane topography. These findings provide a biophysical basis for the ‘ghost fibers’ often observed in TIRF imaging of outer leaflet probes and offer a clearer understanding of how cells maintain large-scale order within a fluid and dynamic lipid bilayer.

## 5. Conclusions

Our study demonstrates that the filamentous ‘ghost’ patterns of GPI-APs observed in TIRF microscopy are a genuine membrane-proximal phenomenon rather than signals originating from the underlying cytoplasm. While the F-actin scaffold is present throughout the sub-membrane space, the spatial templating of outer-leaflet GPI-APs is specifically dependent on membrane cholesterol. This supports a model of cholesterol-mediated transbilayer coupling, where the actin cytoskeleton acts as a spatial scaffold that organizes membrane organization through sparse pinning sites without acting as a rigid barrier to lateral diffusion. These findings provide a biophysical basis for how cells maintain large-scale spatial order while preserving the dynamics of the membrane components.

## Figures and Tables

**Table 1:** Summary of mean diffusion coefficients (*D*), coefficient of variation percentage (CoV%), and sample sizes across penetration depths for F-tractin and GPI.

Depth (nm)	F-Tractin			GPI		
	*D* (μm^2^/s)	CoV (%)	*n*_cells_	*D* (μm^2^/s)	CoV (%)	*n*_cells_
80	0.90±0.21	23.3	10	0.31±0.09	29.0	17
100	0.99±0.10	10.1	6	0.31±0.12	38.7	12
120	1.26±0.21	16.6	5	0.44±0.20	45.4	8
140	1.18±0.33	27.9	6	0.33±0.12	36.3	8
160	1.15±0.24	20.8	8	0.39±0.25	64.1	10

**Table 2:** Diffusion coefficients (*D*) and cell counts (*n*) for F-tractin and GPI under pharmacological treatments.

Condition	F-Tractin *D* (μm^2^/s)	*n*_cells_ (F)	GPI *D* (μm^2^/s)	*n*_cells_ (G)
Resting	0.90±0.21	10	0.31±0.09	17
Lat-A	1.00±0.17	15	0.31±0.08	30
Jas	0.62±0.19	9	0.30±0.06	18
M*β*CD	0.88±0.23	8	0.28±0.09	13

## Data Availability

The original contributions presented in this study are included in the article. Further inquiries can be directed to the corresponding author.
